# A novel approach to estimate the eruptive potential and probability in open conduit volcanoes

**DOI:** 10.1038/srep30471

**Published:** 2016-07-26

**Authors:** Sofia De Gregorio, Marco Camarda

**Affiliations:** 1 Istituto Nazionale di Geofisica e Vulcanologia, sezione di Palermo, via Ugo La Malfa 153, 90146, Palermo, Italy

## Abstract

In open conduit volcanoes, volatile-rich magma continuously enters into the feeding system nevertheless the eruptive activity occurs intermittently. From a practical perspective, the continuous steady input of magma in the feeding system is not able to produce eruptive events alone, but rather surplus of magma inputs are required to trigger the eruptive activity. The greater the amount of surplus of magma within the feeding system, the higher is the eruptive probability.Despite this observation, eruptive potential evaluations are commonly based on the regular magma supply, and in eruptive probability evaluations, generally any magma input has the same weight. Conversely, herein we present a novel approach based on the quantification of surplus of magma progressively intruded in the feeding system. To quantify the surplus of magma, we suggest to process temporal series of measurable parameters linked to the magma supply. We successfully performed a practical application on Mt Etna using the soil CO_2_ flux recorded over ten years.

The evaluation of the eruptive potential and eruptive probability of an active volcano has been one of the most compelling and challenging topic addressed by the volcanology community over the last years. The evaluation of the eruptive potential in open conduit volcanoes is generally based on a constant magma supply rate deduced from long-term series of eruptive rates^1^,^2^,^3^,^4^. The eruptive potential computed as described above gives useful estimations over long timescales (centuries), but this method is less effective when short-term (years or months) estimations are needed because the rate of magma supply can undergo both long-term and short-term changes^1^,^2^,^5^. These changes induce variations on the type and intensity of the eruptive activities of the volcano. The evaluation of the eruptive probability of a volcano can be achieved by two main types of approaches, deterministic and probabilistic. The first is based on the measurement of one or more parameters and the analyses of their variations in response to an eruptive event^6^,^7^. The second includes two different operational procedures, the frequency occurrence procedure and the Bayesian event tree (BET)^8^,^9^,^10^,^11^,^12^,^13^. In the former, the eruptive probability is obtained by means of long-term analyses of eruptive episodes^14^,^15^,^16^,^17^. In the BET, the possible events are organized in a tree-diagram with nodes and branches. The diagram shows all relevant possible outcomes of volcanic unrest at progressively higher degrees of detail. The event tree-diagram is built based on expert consideration of volcanological models, prior knowledge, past data, and monitoring measurements.

These approaches are essentially based on the concept that anomalous changes in measurable parameters indicate the supply of energy/magma and include the intrinsic assumption that any input of magma corresponds to unrest and/or high probability of an eruptive event. However, if this last assumption is valid for closed-conduit volcanoes, where the input of magma is very often a valid trigger of eruptive events, it may be less effective for open conduit volcanoes, where the input of magma occurs continuously. This means that the simple input of magma for open conduit volcanoes does not necessarily lead to a new eruptive phase or unrest. The predisposition of an open conduit volcano to erupt is dependent on the level of magma within the feeding system. The main causes of magma level changes are episodic surplus of magma inputs with respect to the regular supply. Higher magma levels yield a higher probability of surplus of magma input to produce an eruptive event; this means that the surplus of magma has different effects on the system in relation to the amount of magma previously stored inside the feeding system. For this reason, an effective assessment of the eruptive probability must consider the single anomalous variations as well as past changes in the parameter history.

Following this logic, we propose a new way to assess the eruptive potential and eruptive probability in open conduit volcanoes based on the quantification of surplus of magma intruded in the feeding system through time.

## Theoretical Background

In open conduit volcano volatile-rich magma periodically enters the feeding system, and due to buoyancy, ascends along the magmatic column. However, not all of the magma that enters the feeding system of an open conduit volcano is emitted, as deduced by the evidence that the amount of gases emitted by the volcanoes often far exceeds the amount that should be dissolved in the erupted magma^18^,^19^,^20^. A paradox arises when the estimation of the unerupted magma volume is compared with the volumetric capacity of the feeding system; indeed, the unerupted volume always far exceeds the volumetric capacity. To disentangle the paradox, the following two mechanisms are proposed^20^,^21^,^22^,^23^,^24^,^25^,^26^: (i) the existence of magma convection-driven exchange flows in the feeding systems; (ii) gas flushing or percolation from the deep portion of the plumbing system; or the combination of both mechanisms. In the first case, equilibrium between ascending volatile rich magma and descending denser magma is achieved; in the second case, deep exsolved magmatic gas flush and percolate trough the magmatic column; thus, in both cases, new material (magma/gas) is continuously introduced into the feeding system. However, despite the continuous input of material, the eruptive activity of a volcano does not occur steadily; on the contrary, the eruptive activity occurs through eruptive phases, which last for a defined time. From a practical perspective, the continuous steady input of material in the feeding system is not able to produce eruptive events alone, but rather an additional factor is required to trigger the eruptive activity of an open conduit volcano. This additional factor can be a general supply of energy that induces an external release of energy. For a volcanic system, the more effective way to counterbalance the input of energy is the emission of magma, i.e., an eruptive event. The energy supplied to the system has various origins, e.g., enhancement in the magma supply rate, episodic input and/or transfer of surplus of magma, tectonic stress and energy released by seismic swarm. However, the occurrence of the above-mentioned phenomena will not necessarily trigger an eruptive event in the short term because the energy supplies can also be absorbed to the system without being dissipated externally. Whether the system will release the input externally will depend on its energy level and the magnitude of the energy supply. As the energy level increases, the absorption ability of the system decreases. The energy level of the feeding system is related to the amount of magma stored inside it. Indeed, considering the constant capacity of the feeding system, if the amount of magma is greater, less inner space is available, and thus less of the surplus magma/energy can be absorbed. It can be supposed that, at steady state, the regular supply of magma determines an almost constant level of magma/energy in the feeding system. The main factors that can change the level of magma/energy in the system are episodic surplus of magma inputs with respect to the regular supply. It follows that the surplus of magma that occasionally enters into the feeding system represents a supply of material that will sooner or later be removed i.e., it eventually will be emitted. Afterwards, the amount of surplus of magma in the feeding system nearly corresponds to the amount of magma that must be erupted to restore the equilibrium. In this framework, the eruptive potential, understood as the amount of magma that might be emitted, can be estimated by detecting and quantifying the surplus of magma progressively entering into the system. As the amount of surplus of magma stored in the system increases, the system yields higher energy levels as well as increases the system’s propensity for outward dissipation, i.e., its eruptive probability.

## The Methodological Approach

The starting point of the approach is to find a reliable way to detect and quantify the surplus of magma entering the feeding system. To this aim, the first step is to determine a measurable parameter strictly related to magma supply dynamics; e.g., gas flux emitted from summit craters, magmatic gas emissions in peripheral areas, ground deformation, etc. The second step is to identify a background threshold value of the parameter representative of the steady and continuous input of magma/gas in the feeding system. Anomalous values above the threshold can be considered representative of the input of surplus of magma into the feeding system with respect to the normal supply. At this stage, a striking point arises: to quantitatively link the magnitude of anomalies recorded to the volume of surplus of magma intruded into the feeding system. This operation cannot disregard a general and adequate interpretative framework of the variations in relation to the magma supply processes. In light of the interpretative framework, we can find a connection between the size and duration of the anomalies and the total volume of the intruded surplus of magma. Once this goal is achieved, the cumulative curve of the theoretical volume of surplus of magma entering the system can be obtained. At this point, the eruptive potential, over a determinate period of time, is easily computed by the difference of cumulative values and the volume of eruptive products (VEP) progressively emitted. A simple statistical treatment can be applied to the time series of the eruptive potential to highlight periods of high levels of eruptive potential and, in turn, periods with a high eruptive probability.

## Results

### Application to the Mt Etna volcano

**Figure 1:**
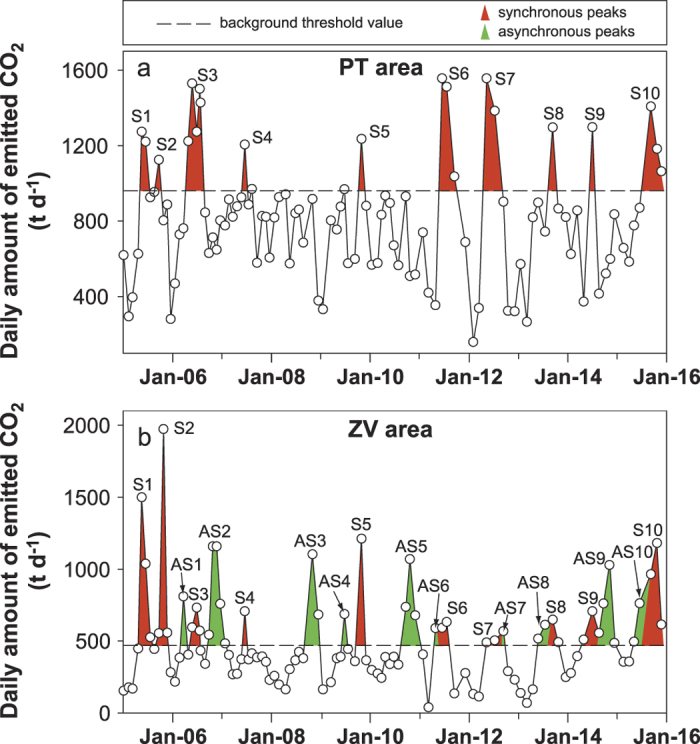
Temporal variations of magmatic CO_2_ emissions between January 2005 and December 2015 in the PT (**a**) and ZV (**b**) areas. The horizontal dashed lines indicate threshold values for each area^28^. Red peaks indicate anomalies recorded almost synchronously in the two areas and identify surplus of magma input in the deep portions of the feeding system (18–10 km b.s.l.). The green peaks indicate anomalies recorded only in the ZV area and indicate magma migration towards the intermediate portions (10–3 km b.s.l.). (The data from 2005–2010 are obtained from Camarda *et al.*^28^; data from 2010 to 2015 are unpublished data obtained and processed as described in Camarda *et al.*^28^).

To test the feasibility of the approach, we performed a practical application by using ten years of soil CO_2_ emission data (published and unpublished), acquired monthly at 130 sites in two peripheral areas of Mt Etna Volcano. The two areas are located approximately 20 km apart to the southwest (PT area) and east (ZV area) with respect to the summit craters (Supplementary Fig. S1). As reported by many authors^27^,^28^,^29^, the CO_2_ emissions in these areas are linked to magma supply dynamics, and anomalous discharges of CO_2_ are attributed to CO_2_ released by surplus of magma intruded at different depths. According to Camarda *et al.*^28^, the measured values of the soil CO_2_ flux can be processed to obtain the amount of magmatic CO_2_ emitted in these areas. In Fig. 1a,b, the temporal variation of the daily magmatic CO_2_ emitted (expressed in t d^−1^) is shown from 2005 to 2015 in the considered areas. In these graphs, we plotted the threshold values to define periods of anomalous release of volcanic CO_2_ relative to the normal volcanic emissions. The threshold values were deduced from changes in the slopes of the relative probability plots^30^. The periods of anomalous degassing (hereafter referred to as peaks) signal the input in the feeding system of surplus of magma. Synchronous peaks are recorded in both areas (shown in red in Fig. 1), whereas peaks recorded only in the ZV area are designated as asynchronous (shown in green in Fig. 1). According to the interpretative framework proposed by Camarda *et al.*^28^, the synchronous peaks identify the input in the deep portions of the feeding system (18–10 km b.s.l.), whereas asynchronous peaks indicate magma migration towards the intermediate portions (10–3 km b.s.l.). In addition to indicating the occurrence of surplus of magma input episodes, the peaks also give an estimation of the amount of magma involved in each episode. In fact, according to Henry’s law, for a determinate amount of dissolved CO_2_, a corresponding mass of magma exists at a determinate depth. To compute the amount of ascending magma associated with the recorded peaks, we followed the expression derived by Harris and Rose^31^ from a mass balance of CO_2_ in a magma body. Table 1 reports the volumes of ascending magma computed for the period of 2005–2015. Because our magma estimation is based only on a limited amount of the entire budget of CO_2_ emissions, our estimations are only a partial estimate of the entire surplus of magma volume that has entered and ascended into the feeding system. The computed volume is one order of magnitude less (10^5^) than the total volume of lava usually involved in the eruptive events, i.e., 10^6^ (Supplementary Table S1). Despite the fact that we only have a partial constraint on the volume of magma involved, it should be noted that is consistent, and hence the magnitude of the recorded changes is proportional to the amount of surplus of magma that entered into the feeding system. A key clue supporting this inference is demonstrated by the fact that the increase in soil CO_2_ flux recorded in the PT and ZV areas before the 1991–1993 eruption^27^ and the associated VEP were one order of magnitude higher than those recorded and emitted during the 2005–2015 period. In conclusion, the partial volume of magma deduced by the area of anomalies recorded is directly proportional to the total volume of magma involved in the magma surplus episodes.

### Conversion factors

**Figure 2:**
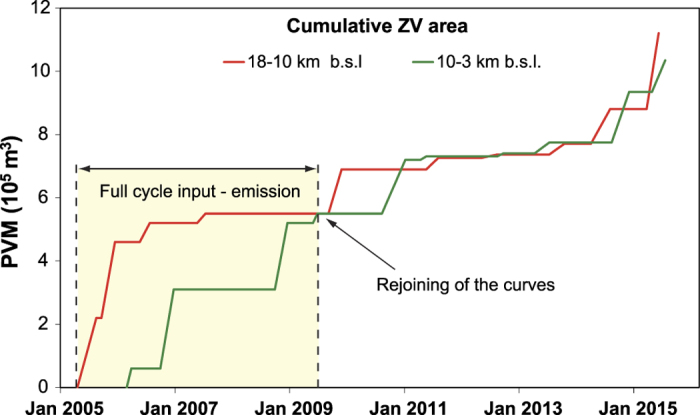
Cumulative curves of the partial volumes of magma (PVM) progressively intruding into the feeding system for ZV area. The red curve is relative to magma intruded in the deep portion of feeding system, and a green curve relative to the intermediate portion. The rejoining of these two curves indicates that all of magma entered in the depth portion was transferred to the intermediate portion.

To obtain a conversion factor able to quantitatively link the estimates of the partial volume of magma with the total volume of surplus of magma, we can compare the partial volume of magma with the VEP, because the surplus of magma corresponds to the amount of magma that eventually will be emitted. The comparison must be done over a “full cycle” of input, transfer of magma and emission of eruptive products. In other words, within the observation period, we must determine an initial phase in which the amount of surplus of magma in the feeding system is negligible, and a final phase in which all of the surplus of magma intruded into the deep portion of the feeding system was emitted. We can assume the beginning of the cycle coincides with the end of the 2004–2005 eruption (March 2005) of the monitoring period because, as shown by several investigations at this time, the feeding system can be considered almost completely “empty”^32^. Thus, it is reasonable to suppose that negligible surplus of magma is present in the feeding system. To identify the end of the *cycle*, we must find a time where all the surplus of magma intruded in the deep portion was emitted. As a first step, we identified a time at which all of the magma that had intruded into deep portion moved towards the intermediate portions. To this end, we used the curve of cumulative volumes of magma intruded at two different depths relative at the ZV area (Fig. 2). In fact, the rejoining of the curves relative to the two different depths indicates that all of the magma that entered into the deep portion is transferred to the intermediate portion of the system. A clear rejoining of the curve is observed at the end of the 2008–2009 eruptive period (July 2009).

We must then ensure that only a negligible amount of magma intruded in the deep portions was not emitted and still remains in the intermediate portions. A guideline to test this condition is the chemical composition of the products emitted during the early events following the 2008–2009 eruption, because it allows us to establish if they derived from magma resident in the feeding system at end of the 2008–2009 eruption or derived from new magma intruded afterwards. The resumption activity at Etna occurred after 18 months, on January 2011 (Supplementary Table S1), with intense explosive activity observed at NCSE, and the products emitted displayed a chemical composition comparable with the 2007 and 2008–2009 products^33^,^34^. This means that the products derived by magma intruded after the end of 2008–2009 eruption because otherwise, theirs chemical composition would have to exhibit more evolved composition with respect to the 2008–2009 products. This implies that only a negligible amount of magma remains in the intermediate portions at the end of 2008–2009. Hence we can properly set as the end of the cycle the end of the 2008–2009 eruptive event.

Once a full cycle of the input and output of surplus of magma is determined, we must find an appropriate value of emitted VEP. In the first instance, we can consider the entire VEP emitted during the above-considered period (from 2005 to 2009); then, we must exclude any volume of magma present in the feeding system before May 2005 (the data of the first input recorded). To this end, we consider the chemical analysis of the eruptive products emitted in the first eruptive event (July–December 2006). According to Nicotera and Viccaro^35^, the products erupted during 15 July–21 October, 2006 can be considered as deriving from drainage of de-hydrated magma already occupying the open-conduit system at least from 2001. Hence, we must consider only a part of VEP emitted in 2006. On the other hand, the emission rate increased notably in conjuntion with the emission of a more basic product after 21 October, and 37 × 10^6^ m^3^ of magma was emitted after this date^4^.

On the basis of VEP reported in the literature (Supplementary Table S1), and considering 37 × 10^6^ m^3^ for the 2006 eruptive event, we obtain a VEP of 113 × 10^6^ m^3^ over the period of 2005–2009. This value must be associated with the partial magma volume intruded over the same period in the feeding system. The value most suitable for partial magma volume is that computed for the PT area because, in this area, the magmatic CO_2_ can only be derived from the deep portions of the feeding system (10–18 km b.s.l.). In contrast, the area of ZV receives the emission of CO_2_ exsolved at different depths, giving a greater degree of uncertainty in the association of the partial magma volume to the VEP. In the PT area over 2005–2009, 4 peaks were recorded that give a total amount of partial magma volume of 0.45 × 10^6^ m^3^; dividing the VEP value by this value, we obtain a conversion factor equal to 251. This factor can be used to transform the partial magma volume values into volumes of surplus of magma intruded in the feeding system, and in turn, the VEP expected to erupt. Hence by means the conversion factor, we transformed the partial magma volumes intruded over the entire study period into surplus of magma volumes (Table 1). To corroborate the validity of the surplus of magma estimates we compared our results with estimates based on SO_2_ emissions. Actually, the amount of SO_2_ emitted by summit craters is usually used to estimate the volumes of magma entering in the feeding system. We used published data of SO_2_ flux recorded at Mt Etna from 2005 to 2011 and in the same way as for the CO_2_ data we computed the areas of the peaks of anomalous periods of emission. We would like to emphasize, however, that obviously we can make only a rough estimate because, as stated above, to perform accurate estimates we need an interpretative framework of SO_2_ variations in relation to the magma supply processes. For the period 2005–2008, we used the graph reported by Salerno *et al.*^36^, over this time span two periods of anomalous release of SO_2_ can be identified, the first lasts from September 2005 to March 2006 and the second is recorded on July–August 2006. From the area of the peaks we obtained a value of surplus of SO_2_ of 56 × 10^4^ t and of 43 × 10^4^ t respectively. For the period 2009–2011 we used data by Patanè *et al.*^37^, in this case we identify four periods of anomalous degassing. The first lasted from October 2009 to February 2010 the others three occurred between late October 2010 and the end of May 2011. Although these periods last longer compared to the previous ones, they displayed increases in emission much more restricted and overall we obtained a total amount of surplus of SO_2_ of 16 × 10^4^ t for the first peak and 14 × 10^4^ t for the others three peaks. Using the total amount of SO_2_ we computed the corresponding volumes of magma according to Allard^18^. For the first two peaks (September 2005–March 2006 and July–August 2006) we estimated overall a volume of surplus of magma of 87 × 10^6^ m^3^ whereas for the others we estimated overall a volume of surplus of magma 26 × 10^6^ m^3^. These results are consistent with our estimates from our CO_2_ data because from January 2005 to the end of July 2006 we computed a total volume of surplus of magma of 103 × 10^6^ m^3^ and for the rest of the period, until May 2011, we computed a value of 26 × 10^6^ m^3^ (Table 1).

### The eruptive potential, eruptive probability and eruptive activity over the period of 2005–2015 at the Mt Etna volcano

**Figure 3:**
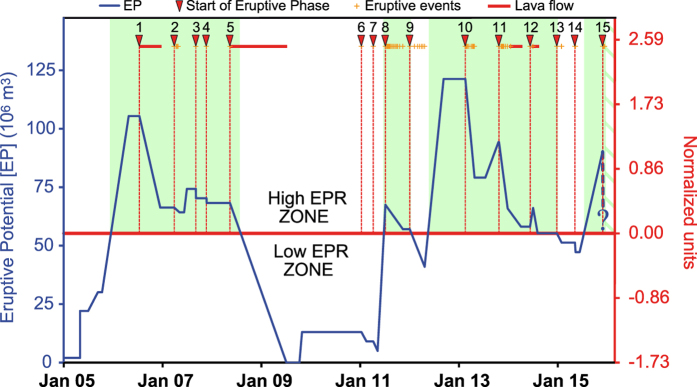
Temporal pattern of the eruptive potential. On the left axis, we reported the absolute values of eruptive potential (EP) in m^3^, and on the right axis the standardized values. The 0 value divides the graph into two zones: one above the 0, corresponding to the high eruptive probability (EPR) zone, and one below the zero, corresponding to the low eruptive probability zone. The intersection between the 0 value (red line) and the curve of the EP defines the periods (light green areas in Fig. 3) with high eruptive probability. The eruptive activity is reported on the top of the graph, and the dotted vertical line marks the initiation of each eruptive phase. We do not have estimates of VEP emitted on December 2015 for this reason at the end the line of eruptive potential is dashed.

To obtain the eruptive potential over time, we must subtract the values of VEP progressively emitted from the values of surplus of magma volumes progressively intruded. In doing so, we obtain the temporal variation of the amount of magma stored in the feeding system that might be potentially erupted (i.e., the eruptive potential of the volcano), expressed in m^3^ (Fig. 3). In Fig.3 the starting value of eruptive potential of 2 × 10^6^ m^3^ corresponds to residual surplus of magma stored in the feeding system after the 2004–2005 eruption^4^,^35^. Each time we recorded a surplus of magma or an eruptive event the curve of eruptive potential was modified according to the volumes of magma entering or emitted (Supplementary Table S2). During the period July 2011–November 2011 we had at the same time the entering of surplus of magma and eruptive activity. Overall the amount of magma entering was less than the amount of eruptive products emitted as result the value obtained at end of this period is lower than starting value of July 2011.

The temporal trend of eruptive potential is quite jagged, alternating with very high values up to 111 × 10^6^, to periods with very low values of 13 × 10^6^. Furthermore, the pattern of surplus of magma displayed two periods with different characteristics. The first period, spanning from 2005 to the middle of 2011, shows a single cycle of increasing/decreasing, with a final low flat signal. In the second period, spanning from the middle of 2011 to the end of the study period, several increasing/decreasing cycles are observed, and the signals do not reach the low values recorded during the first period, but rather fluctuate around a mean value of 50 × 10^6^ m^3^. This general behaviour is consistent with the irregularity and variability recorded in the eruptive activity that occurred during the studied period (reported on top of the graph). Indeed, during 2005–2011, the magma was mainly emitted in two long-lasting large effusive events; in contrast, after 2011, the eruptive activity consists of brief and frequent explosive events and limited lava flow.

Notably, the *magma volume available* inside the feeding system deduced by Viccaro *et al.*^33^ based on petrological, mineralogical, and VEP data, is very similar to our computed EP values. In particular, these authors infer that the *magma volume available* before January 2011 should be less than 18 × 10^6^ m^3^, in agreement with our results for the same period, with an eruptive potential value of 13 × 10^6^ m^3^. In addition, the magma supply dynamics, deduced by petrological and mineralogical data, reported by Behncke *et al.*^34^ and Viccaro *et al.*^33^, perfectly overlap the trend depicted by the eruptive potential line; in fact, both detected significant recharge episodes during the eruptive cycle of 2011. These correspondences prove the reliability of our approach to track and estimate the surplus of magma into the Etnean feeding system.

Once the high reliability of our estimation is demonstrated, we can use the eruptive potential time curve to evaluate the eruptive probability over the considered period. The eruptive probability depends on the amount of surplus of magma stored in the feeding system (i.e. eruptive potential); a higher surplus of magma stored yields a higher eruptive probability. How much higher must the amount of eruptive potential be to be representative of a high probability of an eruptive event occurrence can be established by applying a standardization process to the data (right axis in Fig. 3). In this way, we can divide the graph of eruptive potential into the following two areas: one above the zero, corresponding to the high eruptive probability zone, and one below the zero, corresponding to the low eruptive probability zone. The intersection between the 0 value (red line) and the curve of the eruptive potential define the period (light green areas in Fig. 3) with a high eruptive probability. We identify four periods with a high eruptive probability; the periods have a variable length of time, and the time elapsed from one period to another is also quite variable. In particular, the oscillating pattern near the 0 value displayed by the eruptive potential after 2011 suggests an equilibrium between magma input and eruptive product emission, as argued by Bonaccorso and Calvari^38^ and Behncke *et al.*^34^.

To test the reliability of the method, we counted the number of eruptive phases^4^,^34^,^38^,^39^,^40^,^41^ started during the period of high eruptive probability (we consider multi-events of explosive sequences as single eruptive phase). In total, 12 of 15 eruptive phases started during a period of high eruptive probability, i.e. 80% of the time, the eruptive activity occurred when the eruptive potential was in the high eruptive probability zone. In addition all the events with a VEP > 5 × 10^6^ m^3^ started during a high eruptive probability period.

This result demonstrates that the proposed methodology offers an effective way to evaluate the eruptive probability of an open conduit volcano. An additional but fundamental consideration is that the eruptive potential curve provides a rough estimate of lava volume that might be emitted. This information is fundamentally important in the assessment of volcanic activity evolution.

Finally, despite the fact that we report the CO_2_ flux in the peripheral area as an example, it must be stressed that the new approach is also applicable using other parameters to detect surplus of magma input (e.g., ground deformation data, SO_2_ and CO_2_ plume emissions). Furthermore, from a volcanic risk assessment perspective, anomalous values recorded in a period of high eruptive probability assume a different meaning than those recorded during a period of low eruptive probability. Thus, the new approach can be coupled to canonical methods (e.g., BET methods) to better constrain the weight assigned to the recorded anomalies.

## Methods

A detailed description of soil CO_2_ flux measurement method can be found in Camarda *et al.*^30^. The determination process of partial volume of magma from soil CO_2_ flux is described in Camarda *et al.*^28^.

## Additional Information

**How to cite this article**: De Gregorio, S. and Camarda, M. A novel approach to estimate the eruptive potential and probability in open conduit volcanoes. *Sci. Rep.* **6**, 30471; doi: 10.1038/srep30471 (2016).

## Supplementary Material

Supplementary Information

## Figures and Tables

**Table 1:** Partial volume of magma (PVM) and magma surplus volume (SM).

**PT Area**			
Peak	PVM (10^5^ m^3^)	SM (10^6^ m^3^)	Ref.
S1	0.8	20	[28]
S2	0.3	8	[28]
S3	3	75	[28]
S4	0.4	10	[28]
S5	0.5	13	[28]
S6	3.1	78	[this study]
S7	3.2	80	[this study]
S8	0.6	15	[this study]
S9	0.3	8	[this study]
S10	1.7	43	[this study]

PVM computed by CO_2_ emission following Camarda *et al.*^28^. Magma surplus volume deduced by PVM values using the conversion factor (see text for details).

## References

[b1] Dvorak J. J. & Dzurisin D. Variations in magma supply rate at Kilauea Volcano, Hawaii. J. Geophys. Res. 98, 22255–22268 (1993).

[b2] Andronico D. & Lodato L. Effusive Activity at Mount Etna Volcano (Italy) During the 20th Century: A Contribution to Volcanic Hazard Assessment. Nat. Hazards 36, 407–443 (2005).

[b3] White S. M., Crisp J. A. & Spera F. J. Long-term volumetric eruption rates and magma budgets. Geochem. Geophys. Geosyst. 7, Q03010 (2006).

[b4] Harris A. J. L., Steffke A., Calvari S. & Spampinato L. Thirty years of satellite-derived lava discharge rates at Etna: Implications for steady volumetric output. J. Geophys. Res. 116, B08204 (2011).

[b5] Poland M. P., Miklius A., Sutton A. J. & Thornber C. R. A mantle-driven surge in magma supply to Kīlauea Volcano during 2003–2007. Nat. Geosci. 5, 295–300 (2012).

[b6] Sparks R. S. J. Frontiers: forecasting volcanic eruptions. Earth. Planet. Sci. Lett. 210, 1–15 (2003).

[b7] Terakawa T. *et al.* Monitoring eruption activity using temporal stress changes at Mount Ontake volcano. Nat. Commun. 7, 10797 (2016). 26892716 10.1038/ncomms10797 PMC4762890

[b8] Newhall C. G. & Hoblitt R. P. Constructing event trees for volcanic crisis. B. Volcanol. 64, 3–20 (2002).

[b9] Marzocchi W., Sandri L. & Selva J. BET_EF: a probabilistic tool for long- and short-term eruption forecasting. B. Volcanol. 70, 623–632 (2008).

[b10] Marzocchi W., Sandri L. & Selva J. BET_VH: a probabilistic tool for long-term volcanic hazard assessment. B. Volcanol. 72, 705–716 (2010).

[b11] Brancato A. *et al.* Application of BET_EF at Mount Etna: a retrospective analysis (years 2001–2005). Ann. Geophys. 54, 642–661 (2011).

[b12] Selva J., Marzocchi W., Papale P. & Sandri L. Operational eruption forecasting at high-risk volcanoes: the case of Campi Flegrei, Naples. J. Appl. Volcanol. 1 (2012).

[b13] Sobradelo R., Bartolini S. & Martí J. HASSET: a probability event tree tool to evaluate future volcanic scenarios using Bayesian inference. B. Volcanol. 76, 1–15 (2014).

[b14] Turner M., Cronin S. J., Bebbington M. & Platz T. Developing a probabilistic eruption forecast for dormant volcanoes; a case study from Mt Taranaki, New Zealand. B. Volcanol. 70, 507–515 (2008).

[b15] Dzierma Y. & Wehrmann H. Statistical eruption forecast for the Chilean Southern Volcanic Zone: typical probabilities of volcanic eruptions as baseline for possibly enhanced activity following the large 2010 Concepción earthquake. Nat. Hazards Earth Syst. Sci. 10, 2093–2108 (2010).

[b16] Sandri L., Marzocchi W. & Gasperini P. Some insights on the occurrence of recent volcanic eruption of Mount Etna volcano (Sicily, Italy). Geophys. J. Int. 163, 1203–1218 (2005).

[b17] Marzocchi W. & Zaccarelli L. A quantitative model for the time-size distribution of eruptions. J. Geophys. Res. 111, B0420 (2006).

[b18] Allard P. Endogenous magma degassing and storage at Mount Etna. Geophys. Res. Lett. 24, 2219–2222. (1997).

[b19] Fischer T. P. Fluxes of volatiles (H_2_O, CO_2_, N_2_, Cl, F) from arc volcanoes. Geochem. J. 42, 21–38 (2008).

[b20] Shinohara H. Excess degassing from volcanoes and its role on eruptive and intrusive activity. Rev. Geophys. 46, RG4005 (2008).

[b21] Spilliaert N., Métrich N. & Allard P. S-Cl-F degassing pattern of water-rich alkali basalt: modelling and relationship with eruption styles on Mount Etna volcano. Earth Planet. Sci. Lett. 248, 772–786 (2006).

[b22] Burton M. R., Mader H. M. & Polacci M. The role of gas percolation in quiescent degassing of persistently active basaltic volcanoes. Earth. Planet. Sci. Lett. 271, 123–134 (2007).

[b23] Polacci M., Baker, Don R., Bai L. & Mancini L. Large vesicles record pathways of degassing at basaltic volcanoes. B. Volcanol. 70, 1023–1029 (2008).

[b24] Oppenheimer C., Lomakinab A. S., Kylec P. R., Kingsburyb N. G. & Boichua M. Pulsatory magma supply to a phonolite lava lake. Earth Planet. Sci. Lett. 284, 392–398 (2009).

[b25] Carbone D. & Poland M. P. Gravity fluctuations induced by magma convection at Kilauea Volcano, Hawaii. Geology 40, 803–806 (2012).

[b26] Ferlito C. *et al.* The volatile flushing triggers eruptions at open conduit volcanoes: Evidence from Mount Etna volcano (Italy). Lithos 184–187, 447–455 (2013).

[b27] Federico C., Camarda M., De Gregorio S. & Gurrieri S. Long-term record of CO_2_ degassing along Mt. Etna’s flanks and its relationship with magma dynamics and eastern flank instability. Geochem. Geophys. Geosyst. 12, Q10002 (2011).

[b28] Camarda M., De Gregorio S. & Gurrieri S. Magma-ascent processes during 2005–2009 at Mt Etna inferred by soil CO_2_ emissions in peripheral areas of the volcano. Chem. Geol. 330–331, 218–227 (2012).

[b29] Liuzzo M., Gurrieri S., Giudice G. & Giuffrida G. Ten years of soil CO_2_ continuous monitoring on Mt. Etna: exploring the relationship between processes of soil degassing and volcanic activity. Geochem. Geophys. Geosyst. 14, 2886–2899 (2013).

[b30] Camarda M., Gurrieri S. & Valenza M. CO_2_ flux measurements in volcanic areas using the dynamic concentration method: influence of soil permeability. J. Geophys. Res. 111, B0520 (2006).

[b31] Harris D. M. & Rose W. I. Dynamics of carbon dioxide emissions, crystallization, and magma ascent: hypotheses, theory, and applications to volcano monitoring at Mount St. Helens. B. Volcanol. 58, 163–174 (1996).

[b32] Bonaccorso A., Bonforte A., Guglielmino F., Palano M. & Puglisi G. Composite ground deformation pattern forerunning the 2004–2005 Mount Etna eruption. J. Geophys. Res. 111, B1220 (2006).

[b33] Viccaro M. *et al.* Continuous magma recharge at Mt. Etna during the 2011–2013 period controls the style of volcanic activity and compositions of erupted lavas. Miner. Petrol. 109, 67–83 (2015).

[b34] Behncke B. *et al.* The 2011–2012 summit activity of Mount Etna: Birth, growth and products of the new SE crater. J. Volcanol. Geotherm. Res. 270, 10–21 (2014)

[b35] Nicotra E. & Viccaro M. Transient uprise of gas and gas-rich magma batches fed the pulsating behavior of the 2006 eruptive episodes at Mt. Etna volcano. J. Volcanol. Geotherm. Res. 227–228, 102–118 (2012).

[b36] Salerno G. G. *et al.* Three-years of SO_2_ flux measurements of Mt. Etna using an automated UV scanner array: Comparison with conventional traverses and uncertainties in flux retrieval. J. Volcanol. Geotherm. Res. 183, 76–83 (2009).

[b37] Patanè D. *et al.* Insights into magma and fluid transfer at Mount Etna by a multiparametric approach: A model of the events leading to the 2011 eruptive cycle. J. Geophys. Res. 118, 3519–3539 (2013).

[b38] Bonaccorso A. & Calvari S. Major effusive eruptions and recent lava fountains: balance between expected and erupted magma volumes at Etna volcano. Geophys. Res. Lett. 40, 6069–6073 (2013).

[b39] Andronico D., Cristaldi A. & Scollo S. The 4–5 September 2007 lava fountain at South-East Crater of Mt Etna, Italy. J. Volcanol. Geotherm. Res. 173, 325–328 (2008).

[b40] De Beni, *et al.* The continuing story of Etna’s New Southeast Crater (2012–2014): Evolution and volume calculations based on field surveys and aerophotogrammetry. J. Volcanol. Geotherm. Res. 303, 175–186 (2015).

[b41] INGV - Osservatorio Etneo. *Aggiornamenti sull’attività eruttiva dell*’*Etna* Available at: http://www.ct.ingv.it/it/?option=com_content&view=article&id=1035; http://www.ct.ingv.it/it/?option=com_content&view=article&id=889&catid=102 (2015).

