# Supplementary material for: A novel approach to estimate the eruptive potential and probability in open conduit volcanoes
Source: Sci Rep. 2016 Jul 26;6:30471. doi: 10.1038/srep30471 (PMC4960538; doi:10.1038/srep30471)
Supplement: Supplementary Information [file srep30471-s1.pdf]

Supplementary information

A novel approach to estimate the eruptive potential and probability in open conduit volcanoes

Sofia De Gregorio and Marco Camarda

Istituto Nazionale di Geofisica e Vulcanologia, sezione di Palermo.

Contents

- Supplementary Figure S1.
- Supplementary Table S1.
- Supplementary Table S2.

*Corresponding author: E-mail: sofia.degregorio@ingv.it, Tel: +390916809439, Fax: +39091680944

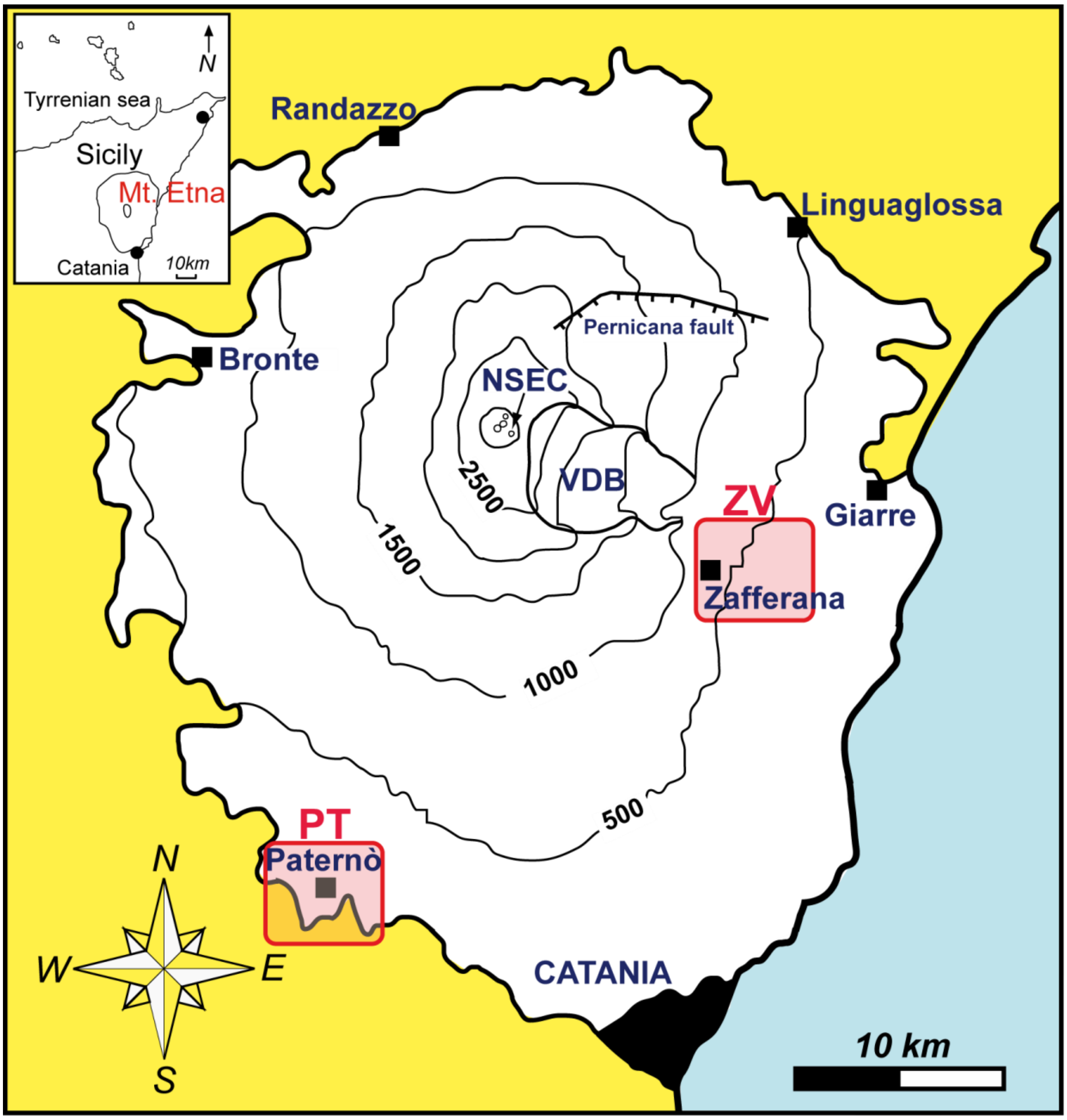

Supplementary Figure S1. Sketch of Mt Etna showing the placement of the areas (red boxes) where we measured the soil CO_2 flux. PT, Paternò area; ZV, Zafferana Etnea; VDB, Valle del Bove; NSEC, New South-Est Crater. This figure was generated using Adobe Illustrator CS3 software.

Eruptive phase	Start / End	VEP × 10^6 (m^3)	Ref.*
1	14/07/2006 / 15/12/2006	39	[4]
2	29/03/2007 / 06/05/2007	2	[4]
3	05/09/2007 / 06/09/2007	4	[39]
4	23/11/2007 / 24/11/2007	2	[38]
5	14/05/2008 / 06/07/2009	68	[4]
6	12/01/2011 / 18/02/2011	4	[34]
7	10/04/2011 / 12/05/2011	4	[34]
8	09/07/2011 / 15/11/2011	26	[34]
9	05/01/2012 / 24/04/2012	16	[34]
10	19/02/2013 / 27/04/2013	42	[40]
11	26/10/2013 / 07/04/2014	36	[40]
12	14/06/2014 / 08/08/2014	11	[40]
13	28/12/2014 / 02/02/2015	4	[41]
14	11/05/2015 / 16/05/2015	2	[41]
15	03/12/2015 / 08/12/2015	?	

Supplementary Table S1. The eruptive phases and the relative VEP. *The references are reported in the main text.

SM	Eruptive Phase		EP (10^6 m^3)
		01/01/2005	2
S1		01/05/2005	2
		30/06/2005	22
S2		10/09/2005	22
		11/10/2005	30
S3		26/04/2006	30
		12/07/2006	105
	1	14/07/2006	105
		15/12/2006	66
	2	29/03/2007	66
		06/05/2007	64
S4		10/06/2007	64
		30/06/2007	74
	3	05/09/2007	74
		06/09/2007	70
	4	23/11/2007	70
		24/11/2007	68
	5	14/05/2008	68
		06/07/2009	0
S5		10/10/2009	0
		30/10/2009	13
	6	12/01/2011	13
		18/02/2011	9
	7	10/04/2011	9
		12/05/2011	5
S6 (I)*		12/05/2011	5
		09/07/2011	67
S6 (II)	8	09/07/2011	67
		15/11/2011	57
	9	05/01/2012	57
		24/04/2012	41
S7		24/04/2012	41
		11/09/2012	121
	10	19/02/2013	121
		27/04/2013	79
S8		17/07/2013	79
		22/10/2013	94
	11**	26/10/2013	94
		29/12/2013	66
		07/04/2014	58
S9		12/06/2014	58
		05/07/2014	66
	12	05/07/2014	66
		08/08/2014	55
	13	28/12/2014	55
		02/02/2015	51
	14	11/05/2015	51
		16/05/2015	47
S10		15/06/2015	47
		02/12/2015	90
	15	02/12/2015	90
		07/12/2015	?

Supplementary Table S2. The table lists the surplus of magma input (SM), the eruptive phases and the relative value of eruptive potential (EP). *****The magma surplus input S6 was separated in two phases 6 (I) and 6 (II) because from 09/07/2011

started also the eruptive phase 8. The phase 6 (I) lasts until the beginning of eruptive activity and in this case we recorded only the increase of the EP. During the Phase 6 (II) we had at the same time the entering of surplus of magma and eruptive activity. In this case we made a balance between input and output. Overall the amount of magma entering was less than the amount of eruptive products emitted as result the value obtained at end of this period is lower than starting value of July 2011. **For the eruptive phase 11 we consider two different magma output rate because from 26/10/2013 to 29/12/2013 the emission of eruptive product occurred mainly by lava fountains after that the emissions occurred by lava flow at low emission rate.
